# Association between dietary diversity and risk of depressive symptoms in Chinese children, adolescents, and college students

**DOI:** 10.3389/fnut.2025.1575351

**Published:** 2026-01-14

**Authors:** Zhongyu Ren, Liya Gao, Zixuan Hao, Boyang Zhao, Jin Li, Guilin Li, Jianhua Cao, Xuemei Tang

**Affiliations:** 1 School of Physical Education, Southwest University, Chongqing, China; 2 Department of Rheumatology and Immunology, Children's Hospital of Chongqing Medical University, Chongqing, China; 3 National Clinical Research Center for Children and Adolescents' Health and Diseases, Chongqing, China; 4 Ministry of Education Key Laboratory of Child Development and Disorders, Chongqing, China; 5 Chongqing Key Laboratory of Child Rare Diseases in Infection and Immunity, Chongqing, China; 6 Children's Hospital of Chongqing Medical University, Chongqing, China; 7 Department of Physical Education, Chongqing Institute of Foreign Studies, Chongqing, China

**Keywords:** dietary diversity, depressive symptoms, children, adolescents, Chinese population

## Abstract

**Background and objective:**

Childhood, adolescence, and emerging adulthood represent critical transitional periods characterized by rapid biological, psychological, and social development, each of which may distinctly influence diet–mood interactions. To date, no study has concurrently examined the association between dietary diversity and depressive symptoms across the full developmental spectrum spanning these life stages. As a result, age-specific vulnerabilities and potential windows for intervention remain poorly understood. Using a large and diverse sample of Chinese children, adolescents, and university students, this cross-sectional study aimed to explore the relationship between dietary diversity and depressive symptoms across these key developmental periods. The findings may help inform the design of targeted, developmentally appropriate nutritional strategies for depression prevention.

**Materials and methods:**

In this cross-sectional investigation, a total of 11,856 Chinese college students and 1,281 children and adolescents were enrolled. All participants completed self-administered questionnaires assessing demographic characteristics, lifestyle behaviors, dietary diversity, and depressive symptoms [evaluated with the Patient Health Questionnaire-9 (PHQ-9) and the 20-item Zung Self-Rating Depression Scale (SDS)]. Multivariable logistic regression analyses were employed to examine the associations between dietary diversity and depressive symptoms, with adjustment for relevant confounding factors.

**Results:**

The prevalence of depressive symptoms was 18.9% (2,245/11,856) among college students and 4.7% (60/1,281) among children and adolescents. Among college students, a significant inverse relationship was observed between dietary diversity scores and depressive symptoms. Compared to participants with a score of 0, the adjusted odds ratios decreased progressively with higher scores, ranging from OR = 0.94 (95% CI: 0.39, 2.30) for a score of 1 to OR = 0.33 (95% CI: 0.13, 0.81) for a score of 9. Similarly, among children and adolescents, higher dietary diversity was associated with markedly lower odds of depressive symptoms, with ORs declining from 0.164 (95% CI: 0.007, 3.837) for one food score to 0.026 (95% CI: 0.002, 0.390) for seven food scores, relative to the zero-score reference group. In analyses of specific food groups, college students showed significant inverse associations between depressive symptoms and consumption of vegetables (OR = 0.67, 95% CI: 0.55, 0.81), fruits (OR = 0.78, 95% CI: 0.70, 0.88), red meat (OR = 0.85, 95% CI: 0.75, 0.95), and soy products (OR = 0.89, 95% CI: 0.80, 0.99). Among children and adolescents, significant associations were observed for multiple dietary factors, with inverse associations for fruit intake (*P* = 0.019) and breakfast consumption (*P* < 0.001), and positive associations for sugar-sweetened beverages (*P* = 0.025), fried foods (*P* < 0.001), fast food (*P* < 0.001), and processed foods (*P* = 0.033).

**Conclusions:**

This study establishes a significant inverse relationship between dietary diversity and depressive symptoms. The results support the integration of dietary diversity into public health recommendations and behavioral interventions. Specifically, fostering diverse and healthy eating patterns emerges as a promising, practical strategy for the prevention of depressive symptoms, underscoring the role of nutrition in mental well-being.

## Introduction

According to the World Health Organization (WHO), depression affects an estimated 280 million people globally, accounting for 3.8% of the world's population and ranking as the fourth most prevalent health disorder worldwide (1). The WHO further projects that by 2030, depression will become the leading cause of global disability (2). Depression, as a chronic and recurrent condition, often requires long-term antidepressant treatment, potentially resulting in significant side effects (3). To address this growing challenge, WHO launched the Mental Health Action Plan 2013–2030, urging countries to implement effective strategies to manage mental health conditions, including depression (4). In China, the lifetime prevalence of depression among adults is approximately 3.4%. Each year, an estimated 280,000 suicide deaths occur, of which 40% are attributed to individuals with depression (5). Notably, students from primary to higher education constitute about half of all people experiencing depressive symptoms (5). Adolescents and young adults are especially susceptible to mental health difficulties due to the complex interplay of biological (6), academic (7), and social stressors (7). Therefore, identifying key risk and protective factors for depressive symptoms in this population is crucial for developing effective prevention and intervention strategies.

Although current evidence indicates a positive association between suboptimal nutrition and adverse mental health outcomes, investigating this relationship specifically in children, adolescents, and university students is critical, as they are in a crucial window for establishing lifelong dietary and mental health patterns. Rising concerns about depressive symptoms among children, adolescents, and young adults parallel notable shifts in their dietary patterns. For example, obesity prevalence during the 2012–2023 period was 1.5 times higher than the rate in 2000 (8). Findings from the China Health and Nutrition Survey (1991–2009) revealed significant changes in the dietary patterns of children and adolescents aged 7–17, showing a clear trend toward increased intake of total fats and cholesterol alongside markedly reduced consumption of dairy products and vegetables (including both dark- and light-colored varieties) (9).

According to data from the 2023 National Statistical Bulletin on Education Development, China's formal education system comprised a total enrollment of 291 million students across all levels (10). This figure comprises 161 million children and adolescents, as well as 47.63 million higher education students (10). However, the predominant focus on weight status in existing research overlooks critical (11), broader diet-related outcomes. Dietary diversity, as a modifiable factor and a holistic measure of diet quality, presents a significant opportunity for public health intervention. Therefore, investigating its connection to the substantial burden of mood disorders in children, adolescents, and college students is not only timely but essential for developing actionable strategies.

Therefore, this study aims to clarify the association between dietary diversity and depressive symptoms in Chinese children, adolescents, and university students. By elucidating this relationship, we seek to strengthen the theoretical foundation for nutritional interventions. The findings could ultimately provide valuable insights for integrating dietary strategies into public health initiatives, thus aiding in the prevention and management of depression in these populations.

## Study setting and period

This study adopted a cross-sectional design. For university students, questionnaires were distributed at physical fitness testing centers located within 11 colleges in August and October 2023, respectively. For children and adolescents, purposive sampling was utilized, and questionnaires were distributed by doctors or teachers to all eligible children and adolescents at Chongqing Medical University Children's Hospital and a primary school in Qijiang District, Chongqing, during the same period.

## Inclusion and exclusion criteria

Eligibility required enrollment in an elementary, junior high, or high school in Chongqing. Participants (or their guardians) and their homeroom teachers were required to provide informed consent after being fully informed of the study's purpose. Additionally, participants needed the capability to complete the survey independently. Exclusion criteria included a history of psychological or other mental illnesses, or any condition that could impede the independent completion of the questionnaire.

## Sample size and sampling procedures

Regarding participant recruitment among college students, the study utilized a stratified random sampling approach that considered both sampling variability and academic grade distribution. Using a random number table, students from one or two grades were randomly chosen across 33 majors from 11 universities. Initially, 12,100 students (1,100 per university) were invited to participate. Participants were excluded based on the following criteria: (1) incomplete or missing data for key variables (dietary behaviors or depressive symptom assessments, *n* = 143); (2) age outside the targeted range (*n* = 51); or (3) implausible or inconsistent responses detected during quality control checks (*n* = 50). After applying these criteria, 11,856 participants remained in the final analysis with a median age of 19.0 years.

For children and adolescents, nonrandom purposive sampling was used to recruit participants from a primary school in Qijiang District and Chongqing Medical University Children's Hospital in Chongqing.

## Variables

Depressive symptoms were the dependent variable, while dietary diversity (or individual food consumption), sex, age, sleep duration, region of origin, ethnicity, BMI, father's education level, mother's education level, grade level and family economic status were independent variables. For school-aged children and adolescents, all data regarding depressive symptoms, dietary behaviors, and demographic information were provided by parents or legal guardians.

### Operational definitions of depressive symptoms

This study used two validated self-report tools to assess depressive symptoms in college students and children and adolescents separately. University students completed the Self-Rating Depression Scale (SDS), which has been widely used in Chinese college populations and demonstrates strong reliability for capturing subclinical depressive symptoms and emotional distress in late adolescents and young adults. School-aged children completed the Patient Health Questionnaire-9 (PHQ-9), a widely recommended screening tool for adolescents due to its brief structure, high diagnostic sensitivity, and suitability for younger respondents.

The selection of these scales was based on their established suitability for the respective age groups. Because the SDS and PHQ-9 measure depressive symptoms using different scoring systems, direct numerical comparisons between university students and school-aged children were avoided. Instead, analyses were conducted separately within each group, focusing on the associations between depressive symptoms and eating behaviors. This approach reduces potential measurement incomparability between the two instruments.

Depressive symptoms in college students were evaluated using the Zung Self-Rating Depression Scale (Zung SDS), which includes 20 items scored on a 4-point Likert scale from 1 (“not at all or very little of the time”) to 4 (“most or all of the time”).

The scale contains ten positively phrased and ten negatively phrased items. The raw scores, which range from 20 to 80, were converted into an index score spanning 25 to 100, where a higher score indicates greater severity of depressive symptoms. The reliability and validity of the Zung SDS for use with Chinese adult populations have been established in previous studies (12). Consistent with prior research, we employed a cutoff score of 50 or above to classify participants as having severe depression (13). The reliability of the scale, assessed using Cronbach's alpha, was 0.847, indicating a high level of internal consistency.

Depressive symptoms in school-aged children and adolescents were assessed using the validated Chinese version of the Patient Health Questionnaire-9 (PHQ-9). This instrument comprises nine items that correspond to DSM-IV depressive symptoms, rated based on their frequency over the preceding 2 weeks. Responses are scored on a 0 (“not at all”) to 3 (“nearly every day”) scale, thereby yielding a total score between 0 and 27, where higher scores indicate greater severity. In this study, the scale demonstrated high internal consistency (Cronbach's α = 0.834). A total score of 10 or above was used as the cutoff to classify participants as having clinically relevant depressive symptoms (14, 15).

### Operational definitions of dietary behavior and dietary diversity

This study assessed dietary diversity among college students based on the recommendations of the Chinese Dietary Guidelines 2022 (16), in conjunction with evidence from previous nationwide studies which indicated that maintaining dietary diversity is associated with increased life expectancy (17). The dietary assessment covered nine food categories: water, eggs, milk and dairy products, vegetables, fruits, red meat, soy products, seafood, and sugar-sweetened beverages.

This study assessed participants' habitual dietary intake through a structured questionnaire. The average daily water consumption was categorized as: < 250 mL/day, 250–499 mL/day, 500–999 mL/day, 1,000–1,499 mL/day, 1,500–2,000 mL/day, or >2,000 mL/day and defined as regular (≥1,500 mL) or irregular (< 1,500 mL) water intake. Egg intake was evaluated based on the average number of eggs consumed per day, with options ranging from none, 1 egg/day, 2 eggs/day, 3 eggs/day, to ≥4 eggs/day and defined as regular (≥1) or irregular (0) consumption. The consumption of milk and dairy products was assessed as average daily volume intake, with response options of never, < 250 mL/day, 250–499 mL/day, 500–750 mL/day, or >750 mL/day and defined as regular (≥250mL) or irregular (< 250mL) consumption. The frequency of vegetable or fruit intake was evaluated based on the average frequency of consumption times per day, with options including never, once per day, twice per day, three times per day, or more than four times per day and defined as regular (once or more) or irregular (never) consumption. The consumption frequency of red meat, soy, and fish intake was assessed as average frequency of consumption, with response options of less than once/never, consume, once, twice, three times, four times, five times, six times, and daily, and defined as regular (twice or more) or irregular (less than twice) consumption. Based on the nine dietary items described above, a binary scoring criterion was applied where a score of 1 was assigned for regular consumption and 0 for irregular consumption, according to the pre-defined criteria for each food item. The scores for all items were then summed to create a total diet diversity score. A higher total score indicates a greater diversity of healthy dietary patterns. Reliability was assessed using intra-class correlation coefficients (ICCs), which ranged between 0.947 and 0.987, reflecting excellent consistency. Detailed information regarding questionnaire items and responses is provided in Supplementary Table 1.

Dietary behaviors among adolescents were assessed using a structured questionnaire. The assessment included seven items focusing on both healthy and unhealthy eating habits: sugar-sweetened beverages, fried food, fruits, vegetables, breakfast consumption, fast food, and processed meats such as salami, bacon, and sausages (18).

The intake frequency was assessed using simplified categorical options. Consumption of potentially less healthy items, specifically sugar-sweetened beverages, fried foods, fast food, and processed meats, was evaluated based on the average number of times consumed per week. Response options included: < 1 time/week, 1, 2, 3, 4, 5, or 6 times/week, or every day. Similarly, breakfast frequency was assessed using the same weekly options. For fruits and vegetables, intake frequency was based on the average number of consumption times per day, with the following options: < 1 time/day, 1 time/day, 2 times/day, or ≥3 times/day. A binary scoring criterion was applied to define regular consumption. For vegetables and fruits, a frequency of once or more per day was considered regular and assigned a score of 1; otherwise, a score of 0 was given. For breakfast, consumption occurring once or more per week was classified as regular and scored 1; otherwise, it was scored 0. For unhealthy food items, including sugar-sweetened beverages, fried foods, fast food, and processed meats, a score of 1 was assigned only in cases of no consumption; any reported consumption was scored 0. A total dietary diversity score was calculated as the sum of all healthy eating behavior scores, with higher composite scores indicating healthier overall dietary patterns. The detailed questionnaire items and corresponding response options can be found in Supplementary Table 2.

### Assessment of relevant covariates

Demographic and lifestyle variables, including sex, age, family economic status, region of origin (Chongqing, Jilin, Liaoning), ethnicity (Han or other), grade level (one or two grades) and sleep duration, were collected from two self-reported questionnaires. Family economic status was assessed using different scales for the two participant groups: for college students, it was categorized into annual income brackets (< 20,000, 20,000–35,000, or >35,000 RMB) (19), whereas for children and adolescents, it was rated subjectively as good, average, or poor (20). Body mass index (BMI) was assessed using standard anthropometric procedures. Trained investigators measured each participant's height and weight following a unified protocol. Height was measured to the nearest 0.1 cm using a portable stadiometer with participants standing barefoot and in an upright position. Weight was measured to the nearest 0.1 kg using a calibrated digital scale, with participants wearing light clothing and no shoes. BMI was then calculated as weight in kilograms divided by height in meters squared (kg/m^2^). In this study, BMI was treated as a continuous variable in the descriptive statistics and regression models.

### Data quality assurance

A rigorous quality assurance protocol was implemented to ensure the accuracy, completeness, and reliability of key variables, including depressive symptoms, dietary diversity, demographic factors, sleep duration, and family economic status. Prior to data collection, all researchers and supervisors underwent a comprehensive two-day training on the data collection tools and procedures. Completed questionnaires were reviewed daily by supervisors to verify completeness and consistency. A large-scale pilot test was conducted in participating schools to evaluate item clarity, response accuracy, and completion time. Standardized and validated instruments, namely the PHQ-9 or SDS and a structured dietary questionnaire, were used to assess depressive symptoms and dietary intake. Demographic and lifestyle data were self-reported via a secure electronic platform that incorporated scope checks, logic validation, and mandatory fields to minimize entry errors and missing data. Daily missing data reports were generated, and targeted deletion was applied when key variables exceeded predefined thresholds. All collected data were anonymized, encrypted, securely stored on servers, and regularly backed up to ensure traceability and integrity for subsequent statistical analysis.

### Data collection procedures

The study employed a cross-sectional design, utilizing the online “Questionnaire Star” platform for data collection. Recruitment and survey distribution were conducted via dedicated WeChat groups administered by school personnel. Following the provision of informed consent, participants completed the questionnaire independently, with an average completion time of 20 min.

### Ethical considerations

Ethical approval for this research was granted by the Ethics Committee of the School of Physical Education at Southwest University. Prior to participation, written informed consent was acquired from all participants; for those under 16 years of age, consent was provided by their legal guardians or next of kin.

### Statistical analysis

Microsoft Excel and IBM SPSS Statistics (Version 27.0) were used for data management and statistical analyses, respectively. Descriptive statistics are presented as medians (IQR) for continuous variables and percentages for categorical variables. Group differences in demographic and lifestyle characteristics, stratified by the presence of depressive symptoms, were evaluated using non-parametric methods.

To control for potential confounders, we constructed three sequential logistic regression models. Model 1 was unadjusted (crude). Model 2 was adjusted for age, gender, parental education, and annual family income. Model 3 included additional adjustment for sleep duration. Model 4 included additional adjustments for region of origin (Chongqing, Jilin, Liaoning), ethnicity (Han or other), BMI, and grade level (one or two grades).

To determine the relative contribution of the nine individual food groups to depressive symptoms, we performed multivariate logistic regression. The analysis yielded odds ratios (ORs) with 95% confidence intervals (CIs) for the association between dietary diversity (independent variable) and depressive symptoms (dependent variable), with statistical significance set at *P* < 0.05.

## Results

### Characteristics of the college student participants

A total of 11,856 respondents (median age: 19.0 years, IQR: 18.0–19.0; 33.2% male) were included in the final analysis. The distribution of annual family income was 35.5% (< 20,000 RMB), 29.9% (20,000–35,000 RMB), and 34.6% (>35,000 RMB). The proportions of fathers and mothers with a primary education or lower, junior high school, high school, and a bachelor's degree or higher were 23.9% vs. 31.9%, 39.5% vs. 36.4%, 21.4% vs. 18.6%, and 15.1% vs. 13.1%, respectively. Adequate sleep duration (6–8 h/night) was reported by 95.6% of the participants.

A total of 2,245 participants (18.9%) reported depressive symptoms. The group with depressive symptoms was significantly older and had a higher proportion of individuals from lower-income households (< 35,000 RMB annually) and with insufficient sleep (all P < 0.001). An elevated prevalence of depressive symptoms was notably associated with increasing age, lower socioeconomic status, and sleep duration outside the 6–8 h range. No other significant intergroup differences were identified (Table 1).

**Table 1:** Characteristics of college students with and without depressive symptoms.

***N* = 11,856**	**Total**	**Categories of depressive symptoms**		***P* value**
		**Without depressive symptoms**	**With depressive symptoms**	
**Sex (male), %**	33.2	33.0	34.1	0.318
**Age, years**	19.0 (18.0, 19.0)	19.0 (18.0, 19.0)	19.0 (18.0, 20.0)	< 0.001
**Annual family income (yuan), %**				
< 20,000	35.5 (4,213)	34.0	42.0	< 0.001
20,000–35,000	29.9 (3,542)	30.4	27.5	
>35,000	34.6 (4,101)	35.6	30.4	
**Father's education level, %**				
Primary and below	23.9 (2,833)	23.9	24.0	0.597
Junior High	39.5 (4,688)	39.8	38.4	
High School	21.4 (2,541)	21.3	22.1	
Bachelor degree and above	15.1 (1,794)	15.0	15.5	
**Mother's education level, %**				
Primary and below	31.9 (3,784)	31.8	32.6	0.060
Junior High	36.4 (4,314)	36.9	34.1	
High School	18.6 (2,207)	18.3	20.0	
Bachelor degree and above	13.1 (1,551)	13.0	13.3	
**Region of origin**				
Chongqing	32.1	35.4	28.7	
Jilin	34.5	30.1	39.1	0.243
Liaoning	33.4	34.6	32.2	
**Ethnicity, %**				
Han Chinese	92.0	92.1	91.8	0.924
**Grade level**				
One grades	51.7	51.2	52.3	0.848
**Sleep duration (6–8 h), %**	95.6 (11,340)	96.7	91.3	< 0.001
**BMI, kg/m** ^**2**^	23.2 (22.5, 23.9)	23.5 (21.9, 24.1)	22.9 (22.1, 23.6)	0.377

### Characteristics of the children and adolescents participants

Participant characteristics are presented in Table 2. The study included 1,281 children and adolescents (53.9% male) with a median age of 10.1 years. Approximately one-quarter of the participants had at least one parent holding a bachelor's degree or higher. Average sleep duration was 8.7 h on weekdays and 9.4 h on weekends. Compared to those without depressive symptoms, participants with depressive symptoms were significantly older (*P* = 0.033), had a lower family economic status (*P* = 0.030), and lower paternal education levels (*P* < 0.001). No other significant differences were observed between the groups (Table 2).

**Table 2:** Characteristics of children and adolescents with and without depressive symptoms.

***N* = 1,281**	**Total**	**Categories of depressive symptoms**		***P* value**
		**Without depressive symptoms**	**With depressive symptoms**	
**Sex (male), %**	53.9	54.3	43.3	0.091
**Age, years**	10.1 (8,11)	9 (8, 11)	10 (8, 12)	0.033
**Family economic condition, %**				
Bad	24.3	23.7	36.7	0.030
Middle	71.7	72.4	56.7	
Good	4.1	3.9	6.7	
**Father's education level, %**				
College degree or above	23.3	23.8	15.0	< 0.001
High school	33.2	33.8	20.0	
Junior high school graduate	33.6	33.3	40.0	
Elementary school graduation	7.8	7.5	15.0	
Elementary school not graduated	1.6	1.3	8.3	
Uneducated or no formal education	0.4	0.3	1.7	
**Mother's education level, %**				
College degree or above	25.2	25.4	21.7	0.001
High school	29.1	29.6	18.3	
Junior high school graduate	32.9	32.8	35.0	
Elementary school graduation	8.9	8.6	15.0	
Elementary school not graduated	3.4	3.3	6.7	
Uneducated or no formal education	0.4	0.2	3.3	
**Sleep duration, hour**				
Sleep time (weekday), h	8.7	9 (8, 9)	9 (8, 9)	0.138
Sleep time (weekend), h	9.4	10 (9, 10)	10 (9, 10)	0.271

### Association between dietary diversity and risk of depressive symptoms in Chinese college students

After adjusting for potential confounders, multivariate logistic regression analysis revealed a significant inverse association between higher dietary diversity scores and the likelihood of depressive symptoms among Chinese college students. Compared to individuals with a dietary diversity score of 0, the adjusted odds ratios (ORs) exhibited a decreasing trend with increasing scores from 0.94 (95% CI: 0.39, 2.30) for a score of 1 to 0.33 (95% CI: 0.13, 0.81) for a score of 9 (*P* for trend < 0.001), as detailed in Table 3.

**Table 3:** Association between combinations of healthy eating behaviors and depressive symptoms in Chinese college students.

***N* = 11,856**	**Number of participants**	**Case of depressive symptoms**	**Model 1**	**Model 2**	**Model 3**	**Model 4**
**Number of healthy eating behaviors**						
0	33	10	1.000 (reference)	1.000 (reference)	1.000 (reference)	1.000 (reference)
1	207	63	1.00 (0.45, 2.23)	1.00 (0.45, 2.23)	0.92 (0.41, 2.10)	0.94 (0.39, 2.30)
2	596	132	0.64 (0.30, 1.39)	0.64 (0.30, 1.39)	0.58 (0.26, 1.28)	0.55 (0.23, 1.22)
3	1,433	281	0.55 (0.26, 1.18)	0.55 (0.26, 1.18)	0.52 (0.24, 1.12)	0.49 (0.20, 1.00)
4	2,216	423	0.53 (0.25, 1.14)	0.53 (0.25, 1.14)	0.49 (0.23, 1.07)	0.43 (0.19, 1.03)
5	2,761	515	0.53 (0.25, 1.12)	0.53 (0.25, 1.12)	0.50 (0.23, 1.07)	0.45 (0.28, 1.02)
6	2,449	439	0.50,(0.23, 1.06)	0.50 (0.23, 1.06)	0.47 (0.22, 1.02)	0.40 (0.18, 1.04)
7	1,554	280	0.51 (0.24, 1.07)	0.51 (0.24, 1.08)	0.47 (0.22, 1.03)	0.40 (0.20, 1.08)
8	507	88	0.48 (0.22, 1.05)	0.48 (0.22, 1.06)	0.46 (0.21, 1.01)	0.38 (0.17, 1.00)
9	100	14	0.37 (0.15, 0.96)	0.37 (0.15, 0.96)	0.35 (0.13, 0.91)	0.33 (0.13, 0.81)
*P*	–	–	< 0.001	< 0.001	< 0.001	< 0.001

Model 1: crude model.

Model 2: adjusted for age, gender, parental educational attainment, and annual family income.

Model 3: Model 2 + sleep duration.

Model 4: Model 3 + region of origin, student ethnicity, BMI, and grade level.

All data are presented as odds ratios (OR) and 95% confidence intervals (CI).

The study also assessed associations between depressive symptoms and engagement in 2–9 dietary behaviors. ORs (95% CI) compared with reference groups (< 2, < 3, < 4, < 5, < 6, < 7) were: 2 behaviors: 0.54 (0.41, 0.72) (*P* < 0.001); 3 behaviors: 0.71 (0.60, 0.84) (*P* < 0.001); 4 behaviors: 0.84 (0.75, 0.94) (*P* = 0.003); 5 behaviors: 0.89 (0.81, 0.98) (*P* = 0.019); 6 behaviors: 0.90 (0.82, 0.99) (*P* = 0.030); 7 behaviors: 0.92 (0.82, 1.05) (*P* = 0.209); 8 behaviors: 0.89 (0.71, 1.11) (*P* = 0.284); 9 behaviors: 0.73 (0.41, 1.29) (*P* = 0.273). Detailed questions appear in Supplementary Tables 3–10.

### Association between individual food consumption and depressive symptoms in Chinese college students

After adjusting for confounders, significant protective associations were observed between the consumption of vegetables, fruits, red meat, soy products, and a reduced likelihood of depressive symptoms among Chinese college students. No significant associations were found for other food items (see Supplementary Tables 11–19).

### Association between combinations of healthy eating behaviors and depressive symptoms in Chinese children and adolescents

Multivariate logistic regression analysis, adjusted for potential confounders, revealed a significant inverse association between higher dietary diversity scores and the likelihood of depressive symptoms among Chinese children and adolescents. Compared to participants with a dietary diversity score of 0, the adjusted odds ratios (ORs) decreased consistently with increasing scores from 0.16 (95% CI: 0.01, 3.84) for a score of 1 to 0.03 (95% CI: 0.00, 0.39) for a score of 7. Complete results are presented in Table 4.

**Table 4:** Association between number of healthy eating behaviors and depressive symptoms in Chinese children and adolescents.

***N* = 1,281**	**Number of participants**	**Case of depressive symptoms**	**Model 1**	**Model 2**	**Model 3**
**Number of healthy eating behaviors**					
0	4	1	1.000 (reference)	1.000 (reference)	1.000 (reference)
1	13	1	0.25 (0.01, 5.26)	0.18 (0.01, 4.185)	0.16 (0.01, 3.84)
2	66	11	0.60 (0.06, 6.32)	0.53 (0.05, 6.091)	0.51 (0.05, 5.75)
3	182	16	0.29 (0.03, 2.94)	0.27 (0.02, 2.907)	0.25 (0.02, 2.70)
4	228	10	0.14 (0.01, 1.44)	0.12 (0.01, 1.396)	0.12 (0.01, 1.35)
5	279	12	0.14 (0.01, 1.4)	0.12 (0.01, 1.320)	0.11 (0.01, 1.25)
6	292	7	0.07 (0.01, 0.78)	0.07 (0.01, 0.86)	0.07 (0.01, 0.81)
7	217	2	0.03 (0.00, 0.40)	0.03 (0.00, 0.41)	0.03 (0.00, 0.39)
*P* for trend		—	< 0.001	< 0.001	< 0.001

Model 1: crude model.

Model 2: adjusted for age and gender.

Model 3: Model 2 + parental educational attainment, and annual family income.

Model 4: Model 3 + sleep duration.

All data are presented as odds ratios (OR) and 95% confidence intervals (CI).

This study also investigated the association between the number of healthy dietary behaviors (ranging from 1 to 7) and depressive symptoms. Compared to the reference group (0 behaviors), individuals engaging in a greater number of healthy dietary behaviors exhibited progressively lower odds of depression in the adjusted model. The adjusted ORs were as follows: 1 behavior: OR = 0.469 (*P* = 0.344); 2 behaviors: OR = 0.240 (*P* < 0.001); 3 behaviors: OR = 0.271 (*P* < 0.001); 4 behaviors: OR = 0.339 (*P* < 0.001); 5 behaviors: OR = 0.284 (*P* = 0.001); 6 behaviors: OR = 0.174 (*P* = 0.016); and 7 behaviors: OR = 0.130 (*P* = 0.084). Detailed results are provided in Supplementary Table 20.

### Association between individual food consumption and depressive symptoms in Chinese children and adolescents

After adjustment for relevant confounders, several individual food consumption behaviors were significantly associated with depressive symptoms in Chinese children and adolescents. Specifically, significant inverse associations were observed with fruit consumption (*P* = 0.019) and regular breakfast habits (*P* < 0.001). In contrast, higher consumption of sugar-sweetened beverages (*P* = 0.025), fried foods (*P* < 0.001), fast foods (*P* < 0.001), and processed foods (*P* = 0.033) was positively associated with depressive symptoms. No statistically significant associations were detected for the remaining food items examined. Detailed results are provided in Supplementary Tables 21–27.

## Discussion

This population-based, cross-sectional study aimed to assess the relationship between dietary diversity and depressive symptoms among Chinese school-aged children, adolescents, and university students. The results revealed a robust inverse association, indicating that higher dietary diversity is associated with a reduced risk of depressive symptoms, consistent with a protective role. Adherence to diverse dietary patterns may thus support mental health and overall well-being across these key developmental periods.

These findings are consistent with previous research conducted in both Chinese and Western populations. For example, multiple cross-sectional studies of Chinese older adults have also reported an inverse association between dietary diversity and depressive symptoms (21–23). This inverse association is further supported by other cross-sectional (24) and retrospective cohort studies (25, 26), particularly those focused on adult women in China. Additionally, dietary diversity and depressive symptoms may exhibit a bidirectional relationship. Similar findings were also identified in Western populations (27–29). This study contributes to the literature by examining dietary patterns in Chinese student populations, identifying dietary diversity as a promising modifiable target for depression prevention strategies.

Oxidative stress results from an imbalance between oxidative processes and antioxidant defenses, producing excess oxidative intermediates (30). Several studies report links between depression and oxidative stress (31). Depressed individuals exhibit increased oxidative stress and reduced antioxidant capacity, as evidenced by decreased plasma antioxidant levels and antioxidant enzyme activity (32, 33). Foods are the primary sources of antioxidants, directly impacting antioxidant capacity. Plant-based foods such as fruits, vegetables, olive oil, and nuts are especially rich in antioxidants (34). Amino acids derived from foods including meat, vegetables, whole grains, eggs, and yogurt serve as key precursors of endogenous antioxidants, such as glutathione peroxidase (GPx) (35). In a two-year randomized controlled trial, adherence to a Mediterranean diet, rich in these foods, reduced oxidative stress markers like 8-iso-PGF2α (36). Population-based research consistently demonstrates that greater intake of antioxidant-rich foods is associated with elevated total antioxidant capacity (TAC) and lower concentrations of malondialdehyde (MDA), a recognized marker of oxidative damage (37). These observations support the proposed role of antioxidants in mitigating oxidative stress.

Depression is a chronic inflammatory disorder characterized by cell-mediated immune activation and a compensatory anti-inflammatory response (38). Various foods, including vegetables, fruits, milk, and dairy products, contain anti-inflammatory compounds (39). These nutrients exhibit potent anti-inflammatory effects (40).

Serotonin deficiency contributes to depression's pathophysiology, as evidenced by reduced plasma serotonin levels in depressed individuals (41). Tryptophan converts to 5-hydroxytryptophan, subsequently forming serotonin, thereby increasing serotonin levels in the brain and alleviating depressive symptoms (42). Previous observational studies identified a significant inverse association between dietary tryptophan intake and depression risk (43). Foods evaluated in this study, such as eggs and seafood, are rich in tryptophan, potentially reducing depression risk.

Despite these significant contributions, several limitations should be noted. First, dietary assessment relied on consumption frequency rather than a standardized Food Frequency Questionnaire (FFQ), potentially limiting generalizability. However, previous research suggests dietary diversity measurements are appropriate due to simplicity. Second, the study population is not fully representative of all Chinese children, adolescents, and college students. Future nationwide and cross-cultural studies should further clarify this association. Third, the cross-sectional design precludes establishing causality between dietary diversity and depressive symptoms. Therefore, prospective cohort studies and randomized controlled trials are needed to explore causal relationships. Fourth, significant regional variations exist in dietary patterns and food preparation methods across China. Future studies should evaluate these regional differences and their potential influence on dietary associations with depressive symptoms. Future research should evaluate regional differences and their potential impact on the associations between dietary behaviors and depressive symptoms. Additionally, although the scales employed (SDS for university students and PHQ-9 for school-aged children) are validated and widely used in their respective populations, the use of different instruments may introduce measurement heterogeneity and limit the direct comparability of depressive symptoms between the two groups. However, as analyses in this study focused on within-group associations rather than cross-group comparisons, this issue minimally affects the validity of the findings. Future studies employing a unified measurement tool across age groups would further enhance comparability. Finally, although dietary diversity was assessed using questions developed in accordance with the Chinese Dietary Guidelines, we did not collect information on the food environment or cafeteria provisions at the participating universities. As a result, it was not possible to examine potential differences in food availability across institutions, which may have led to unmeasured confounding factors.

## Conclusion

This study establishes a significant inverse relationship between dietary diversity and depressive symptoms. The results support the integration of dietary diversity into public health recommendations and behavioral interventions. Specifically, fostering diverse and healthy eating patterns emerges as a promising, practical strategy for the prevention of depressive symptoms, underscoring the role of nutrition in mental well-being.

## Data Availability

The raw data supporting the conclusions of this article will be made available by the authors, without undue reservation.
