# Supplementary material for: Association between dietary diversity and risk of depressive symptoms in Chinese children, adolescents, and college students
Source: Front Nutr. 2026 Jan 14;12:1575351. doi: 10.3389/fnut.2025.1575351 (PMC12846944; doi:10.3389/fnut.2025.1575351)
Supplement: Supplementary file 1 [file Data_Sheet_1.pdf]

Supplement table 1 Description of each item and option for dietary behavior among college students

Item	Questions and Options	
quantity of water	question	How much water do you consume on average per day?
	options	☐less than 250ml<br>☐250-500ml<br>☐500-1000ml<br>☐1000-1500ml<br>☐1500-2000ml<br>☐more than 2000ml
quantity of egg	question	How many eggs do you eat on average per day?
	options	☐never<br>☐one egg<br>☐two eggs<br>☐three eggs<br>☐four or more eggs
quantity of milk and milk products	question	How much milk and milk products do you consume on average per day?
	options	☐never<br>☐less than 250ml<br>☐250-500ml<br>☐500-7500ml<br>☐more than 750ml
quantity of vegetables	question	How frequency do you consume vegetables on average per day?
	options	☐never<br>☐once<br>☐twice<br>☐three times<br>☐more than four times
quantity of fruits	question	How frequency do you consume fruits on average per day?

	options	☐never<br>☐once<br>☐twice<br>☐three times<br>☐more than four times
quantity of red meat	question	How frequency do you consume red meat (including pork, beef, lamb, and processed products such as bacon and sausages) on average per week?
	options	☐never<br>☐once<br>☐twice<br>☐three times<br>☐ four times<br>☐ five times<br>☐ six times<br>☐ every day
quantity of soy and soy products	question	How frequency do you consume soy products on average per week?
	options	☐never<br>☐once<br>☐twice<br>☐three times<br>☐four times<br>☐five times<br>☐six times<br>☐every day
quantity of seafood	question	How frequency do you consume seafood on average per week?
	options	☐never<br>☐once<br>☐twice<br>☐three times<br>☐four times<br>☐five times<br>☐six times

		☐every day
quantity of sugar-sweetened beverages	question	How many sugar-sweetened beverages do you consume on average per day?
	options	☐never<br>☐less than 500ml<br>☐500-1000ml<br>☐1000-1500ml<br>☐more than 1500ml

Supplement table 2 description of each item and option for dietary behavior among adolescents

Item	Question	Option
Sugar-sweetened beverages	How many times sugar-sweetened beverages do you consume on average per week?	<1 time/week
		1 time / week
		2 time / week
		3 time / week
		4 time / week
		5 time / week
		6 time / week
		Everyday
fried food	How many times fried food does you consume on average per week?	<1 time/week
		1 time / week
		2 time / week
		3 time / week
		4 time / week
		5 time / week
		6 time / week
		Everyday
Fruits	How many times fruits do you consume on average per day?	<1 time/day
		1 time/day

		2 time/day
		≥3 time/day
Vegetables	How many times vegetable do you consume on average per day?	<1 time/day
		1 time/day
		2 time/day
		≥3 time/day
Breakfast	How many times breakfast do you consume on average per week?	<1 time/week
		1 time / week
		2 time / week
		3 time / week
		4 time / week
		5 time / week
		6 time / week
		Everyday
Fast food	How many times fast food does you consume on average per week?	<1 time/week
		1 time / week
		2 time / week
		3 time / week

		4 time / week
		5 time / week
		6 time / week
		Everyday
Processed food (salami, bacon, sausages, etc.)	How many times processed foods do you consume on average per week?	<1 time/week
		1 time / week
		2 time / week
		3 time / week
		4 time / week
		5 time / week
		6 time / week
		Everyday

Supplement table 3 Multivariate logistic regression of the risk of depressive symptoms according to healthy eating behaviors (n≥2 vs <2)(Chinese college students)

	Number of participants	Case of depressive symptoms	Model 1 [a]	Model 2 [b]	Model 3 [c]
Healthy eating behaviors (≥=2 vs <2)					
<2	240	73	1.000 (reference)	1.000 (reference)	1.000 (reference)
≥=2	11616	2172	0.53 (0.40, 0.70)	0.53 (0.40, 0.70)	0.54 (0.41, 0.72)
P for trend [d]	—	—	<0.001	<0.001	<0.001

[a] Model 1: crude

[b] Model 2: Adjusted for sex, age, annual family income, father and mother's education (primary and below, junior high, high school, or junior college and above).

[c] Model 3: Model 2+sleep duration and sleep quality.

[d] Adjusted data are expressed as odds ratio (95% confidence intervals).

[e] P for trend were obtained using multivariate logistic regression analyses

Supplement table 4 Multivariate logistic regression of the risk of depressive symptoms according to healthy eating behaviors (n≥3 vs <3)(Chinese college students)

	Number of participants	Case of depressive symptoms	Model 1 [a]	Model 2 [b]	Model 3 [c]
Healthy eating behaviors (≥=3 vs <3)					
<3	836	205	1.000 (reference)	1.000 (reference)	1.000 (reference)
≥=3	11020	2040	0.70 (0.59, 0.82)	0.71 (0.60, 0.83)	0.71 (0.60, 0.84)
P for trend [d]	—	—	<0.001	<0.001	<0.001

[a] Model 1: crude

[b] Model 2: Adjusted for sex, age, annual family income, father and mother's education (primary and below, junior high, high school, or junior college and above).

[c] Model 3: Model 2+sleep duration and sleep quality.

[d] Adjusted data are expressed as odds ratio (95% confidence intervals).

Supplement table 5 Multivariate logistic regression of the risk of depressive symptoms according to healthy eating behaviors (n≥4 vs <4)(Chinese college students)

	Number of participants	Case of depressive symptoms	Model 1 [a]	Model 2 [b]	Model 3 [c]
Healthy eating behaviors (≥=4 vs <4)					
<4	2269	486	1.000 (reference)	1.000 (reference)	1.000 (reference)
≥=4	9587	1759	0.82 (0.74, 0.92)	0.83 (0.75, 0.94)	0.84 (0.75, 0.94)
P for trend [d]	—	—	<0.001	0.002	0.003

[a] Model 1: crude

[b] Model 2: Adjusted for sex, age, annual family income, father and mother's education (primary and below, junior high, high school, or junior college and above).

[c] Model 3: Model 2+sleep duration and sleep quality.

[d] Adjusted data are expressed as odds ratio (95% confidence intervals).

Supplement table 6 Multivariate logistic regression of the risk of depressive symptoms according to healthy eating behaviors (n≥5 vs <5)(Chinese college students)

	Number of participants	Case of depressive symptoms	Model 1 [a]	Model 2 [b]	Model 3 [c]
Healthy eating behaviors (≥=5 vs <5)					
<5	4485	909	1.000 (reference)	1.000 (reference)	1.000 (reference)
≥=5	7371	1336	0.87 (0.79, 0.96)	0.88 (0.80, 0.97)	0.89 (0.81, 0.98)
P for trend [d]	—	—	0.004	0.010	0.019

[a] Model 1: crude

[b] Model 2: Adjusted for sex, age, annual family income, father and mother's education (primary and below, junior high, high school, or junior college and above).

[c] Model 3: Model 2+sleep duration and sleep quality.

[d] Adjusted data are expressed as odds ratio (95% confidence intervals).

Supplement table 7 Multivariate logistic regression of the risk of depressive symptoms according to healthy eating behaviors (n≥6 vs <6)(Chinese college students)

	Number of participants	Case of depressive symptoms	Model 1 [a]	Model 2 [b]	Model 3 [c]
Healthy eating behaviors (≥=6 vs <6)					
<6	7246	1424	1.000 (reference)	1.000 (reference)	1.000 (reference)
≥=6	4610	821	0.89 (0.81, 0.97)	0.89 (0.81, 0.98)	0.90 (0.82, 0.99)
P for trend [d]	—	—	0.013	0.021	0.030

[a] Model 1: crude

[b] Model 2: Adjusted for sex, age, annual family income, father and mother's education (primary and below, junior high, high school, or junior college and above).

[c] Model 3: Model 2+sleep duration and sleep quality.

[d] Adjusted data are expressed as odds ratio (95% confidence intervals).

Supplement table 8 Multivariate logistic regression of the risk of depressive symptoms according to healthy eating behaviors (n≥7 vs <7)(Chinese college students)

	Number of participants	Case of depressive symptoms	Model 1 [a]	Model 2 [b]	Model 3 [c]
Healthy eating behaviors (≥=7 vs <7)					
<7	9695	1863	1.000 (reference)	1.000 (reference)	1.000 (reference)
≥=7	2161	382	0.90 (0.80, 1.02)	0.92 (0.81, 1.04)	0.92 (0.82, 1.05)
P for trend [d]	—	—	0.099	0.159	0.209

[a] Model 1: crude

[b] Model 2: Adjusted for sex, age, annual family income, father and mother's education (primary and below, junior high, high school, or junior college and above).

[c] Model 3: Model 2+sleep duration and sleep quality.

[d] Adjusted data are expressed as odds ratio (95% confidence intervals).

Supplement table 9 Multivariate logistic regression of the risk of depressive symptoms according to healthy eating behaviors (n≥8 vs <8)(Chinese college students)

	Number of participants	Case of depressive symptoms	Model 1 [a]	Model 2 [b]	Model 3 [c]
Healthy eating behaviors (≥=8 vs <8)					
<8	11249	2143	1.000 (reference)	1.000 (reference)	1.000 (reference)
≥=8	607	102	0.86 (0.69, 1.07)	0.87 (0.70, 1.08)	0.89 (0.71, 1.11)
P for trend [d]	—	—	0.169	0.199	0.284

[a] Model 1: crude

[b] Model 2: Adjusted for sex, age, annual family income, father and mother's education (primary and below, junior high, high school, or junior college and above).

[c] Model 3: Model 2+sleep duration and sleep quality.

[d] Adjusted data are expressed as odds ratio (95% confidence intervals).

Supplement table 10 Multivariate logistic regression of the risk of depressive symptoms according to healthy eating behaviors (n≥9 vs <9)(Chinese college students)

	Number of participants	Case of depressive symptoms	Model 1 [a]	Model 2 [b]	Model 3 [c]
Healthy eating behaviors (≥=9 vs <9)					
<9	11756	2231	1.000 (reference)	1.000 (reference)	1.000 (reference)
≥=9	100	14	0.70 (0.39, 1.23)	0.70 (0.40, 1.24)	0.73 (0.41, 1.29)
P for trend [d]	—	—	0.208	0.22	0.273

[a] Model 1: crude

[b] Model 2: Adjusted for sex, age, annual family income, father and mother's education (primary and below, junior high, high school, or junior college and above).

[c] Model 3: Model 2+sleep duration and sleep quality.

[d] Adjusted data are expressed as odds ratio (95% confidence intervals).

Supplement table 11 Multivariate logistic regression of the risk of depressive symptoms according to vegetable consumption (Chinese college students)

	Number of participants	Case of depressive symptoms	Model 1 [a]	Model 2 [b]	Model 3 [c]
Vegetable consumption					
Unhealthy consumption	635	161	1.000 (reference)	1.000 (reference)	1.000 (reference)
Healthy consumption	11221	2084	0.67 (0.56, 0.81)	0.67 (0.55, 0.80)	0.67 (0.55, 0.81)
P for trend [d]	—	—	<0.001	<0.001	<0.001

[a] Model 1: crude

[b] Model 2: Adjusted for sex, age, annual family income, father and mother's education (primary and below, junior high, high school, or junior college and above).

[c] Model 3: Model 2+sleep duration and sleep quality.

[d] Adjusted data are expressed as odds ratio (95% confidence intervals).

Supplement table 12 Multivariate logistic regression of the risk of depressive symptoms according to fruits consumption (Chinese college students)

	Number of participants	Case of depressive symptoms	Model 1 [a]	Model 2 [b]	Model 3 [c]
Fruits consumption					
Unhealthy consumption	2306	512	1.000 (reference)	1.000 (reference)	1.000 (reference)
Healthy consumption	9550	1733	0.78 (0.70, 0.87)	0.78 (0.70, 0.87)	0.78 (0.70, 0.88)
P for trend [d]	—	—	<0.001	<0.001	<0.001

[a] Model 1: crude

[b] Model 2: Adjusted for sex, age, annual family income, father and mother’s education (primary and below, junior high, high school, or junior college and above).

[c] Model 3: Model 2+sleep duration and sleep quality.

[d] Adjusted data are expressed as odds ratio (95% confidence intervals).

Supplement table 13 Multivariate logistic regression of the risk of depressive symptoms according to meat consumption (Chinese college students)

	Number of participants	Case of depressive symptoms	Model 1 [a]	Model 2 [b]	Model 3 [c]
Meat consumption					
Unhealthy consumption	2191	466	1.000 (reference)	1.000 (reference)	1.000 (reference)
Healthy consumption	9665	1779	0.84 (0.75, 0.94)	0.84 (0.75, 0.95)	0.85 (0.75, 0.95)
P for trend [d]	—	—	0.002	0.004	0.005

[a] Model 1: crude

[b] Model 2: Adjusted for sex, age, annual family income, father and mother's education (primary and below, junior high, high school, or junior college and above).

[c] Model 3: Model 2+sleep duration and sleep quality.

[d] Adjusted data are expressed as odds ratio (95% confidence intervals).

Supplement table 14 Multivariate logistic regression of the risk of depressive symptoms according to soy and soy product consumption

(Chinese college students)

	Number of participants	Case of depressive symptoms	Model 1 [a]	Model 2 [b]	Model 3 [c]
Soy and soy product consumption					
Unhealthy consumption	3011	619	1.000 (reference)	1.000 (reference)	1.000 (reference)
Healthy consumption	8845	1626	0.87 (0.79, 0.97)	0.88 (0.79, 0.98)	0.89 (0.80, 0.99)
P for trend [d]	—	—	0.009	0.016	0.026

[a] Model 1: crude

[b] Model 2: Adjusted for sex, age, annual family income, father and mother's education (primary and below, junior high, high school, or junior college and above).

[c] Model 3: Model 2+sleep duration and sleep quality.

[d] Adjusted data are expressed as odds ratio (95% confidence intervals).

Supplement table 15 Multivariate logistic regression of the risk of depressive symptoms according to seafood consumption (Chinese college students)

	Number of participants	Case of depressive symptoms	Model 1 [a]	Model 2 [b]	Model 3 [c]
Seafood consumption					
Unhealthy consumption	7151	1348	1.000 (reference)	1.000 (reference)	1.000 (reference)
Healthy consumption	4705	897	1.01 (0.92, 1.11)	1.01 (0.92, 1.11)	1.01 (0.92, 1.12)
P for trend [d]	—	—	0.771	0.784	0.781

[a] Model 1: crude

[b] Model 2: Adjusted for sex, age, annual family income, father and mother's education (primary and below, junior high, high school, or junior college and above).

[c] Model 3: Model 2+sleep duration and sleep quality.

[d] Adjusted data are expressed as odds ratio (95% confidence intervals).

Supplement table 16 Multivariate logistic regression of the risk of depressive symptoms according to milk consumption (Chinese college students)

	Number of participants	Case of depressive symptoms	Model 1 [a]	Model 2 [b]	Model 3 [c]
Milk consumption					
Unhealthy consumption	8808	1699	1.000 (reference)	1.000 (reference)	1.000 (reference)
Healthy consumption	3048	546	0.91 (0.82, 1.02)	0.93 (0.84, 1.04)	0.94 (0.84, 1.04)
P for trend [d]	—	—	0.095	0.194	0.226

[a] Model 1: crude

[b] Model 2: Adjusted for sex, age, annual family income, father and mother's education (primary and below, junior high, high school, or junior college and above).

[c] Model 3: Model 2+sleep duration and sleep quality.

[d] Adjusted data are expressed as odds ratio (95% confidence intervals).

Supplement table 17 Multivariate logistic regression of the risk of depressive symptoms according to egg consumption (Chinese college students)

	Number of participants	Case of depressive symptoms	Model 1 [a]	Model 2 [b]	Model 3 [c]
Egg consumption					
Unhealthy consumption	5234	1034	1.000 (reference)	1.000 (reference)	1.000 (reference)
Healthy consumption	6622	1211	0.91 (0.83, 1.00)	0.92 (0.84, 1.01)	0.93 (0.84, 1.02)
P for trend [d]	—	—	0.043	0.073	0.114

[a] Model 1: crude

[b] Model 2: Adjusted for sex, age, annual family income, father and mother's education (primary and below, junior high, high school, or junior college and above).

[c] Model 3: Model 2+sleep duration and sleep quality.

[d] Adjusted data are expressed as odds ratio (95% confidence intervals).

Supplement table 18 Multivariate logistic regression of the risk of depressive symptoms according to sugar-sweetened beverages consumption

(Chinese college students)

	Number of participants	Case of depressive symptoms	Model 1 [a]	Model 2 [b]	Model 3 [c]
Sugar-sweetened beverages consumption					
Unhealthy consumption	8379	1577	1.000 (reference)	1.000 (reference)	1.000 (reference)
Healthy consumption	3477	668	1.03 (0.93, 1.13)	1.03 (0.93, 1.14)	1.04 (0.94, 1.15)
P for trend [d]	—	—	0.621	0.538	0.468

[a] Model 1: crude

[b] Model 2: Adjusted for sex, age, annual family income, father and mother's education (primary and below, junior high, high school, or junior college and above).

[c] Model 3: Model 2+sleep duration and sleep quality.

[d] Adjusted data are expressed as odds ratio (95% confidence intervals).

Supplement table 19 Multivariate logistic regression of the risk of depressive symptoms according to water consumption (Chinese college students)

	Number of participants	Case of depressive symptoms	Model 1 [a]	Model 2 [b]	Model 3 [c]
Water consumption					
Unhealthy consumption	10094	1928	1.000 (reference)	1.000 (reference)	1.000 (reference)
Healthy consumption	1762	317	0.93 (0.82, 1.06)	0.93 (0.82, 1.06)	0.94 (0.82, 1.07)
P for trend [d]	—	—	0.273	0.285	0.325

[a] Model 1: crude

[b] Model 2: Adjusted for sex, age, annual family income, father and mother’s education (primary and below, junior high, high school, or junior college and above).

[c] Model 3: Model 2+sleep duration and sleep quality.

[d] Adjusted data are expressed as odds ratio (95% confidence intervals).

Supplement table 20 Multivariate logistic regression of the risk of depressive symptoms according to healthy eating behaviors (Chinese children and adolescents)

	Model 1 a	Model 2 b	Model 3 c
Healthy eating behaviors (≥=1 vs <1)			
<1	1.000 (reference)	1.000 (reference)	1.000 (reference)
≥=1	2.77 (0.619, 12.410)	0.440 (0.091, 2.123)	0.469 (0.098, 2.250)
P for trend d	0.182	0.306	0.344
Healthy eating behaviors (≥=2 vs <2)			
<2	1.000 (reference)	1.000 (reference)	1.000 (reference)
≥=2	4.548 (2.351, 8.798)	0.235 (0.118, 0.467)	0.240 (0.120, 0.480)
P for trend d	< 0.001	< 0.001	< 0.001
Healthy eating behaviors (≥=3 vs <3)			
<3	1.000 (reference)	1.000 (reference)	1.000 (reference)
≥=3	0.256 (0.151, 0.433)	0.266 (0.155, 0.455)	0.271 (0.157, 0.467)
P for trend d	< 0.001	< 0.001	< 0.001
Healthy eating behaviors (≥=4 vs <4)			
<4	1.000 (reference)	1.000 (reference)	1.000 (reference)
≥=4	0.319 (0.185, 0.549)	0.337 (0.194, 0.586)	0.339 (0.195, 0.590)
P for trend d	< 0.001	< 0.001	< 0.001

Healthy eating behaviors (≥=5 vs <5)			
<5	1.000 (reference)	1.000 (reference)	1.000 (reference)
≥=5	0.254 (0.124, 0.522)	0.282 (0.136, 0.584)	0.284 (0.137, 0.589)
P for trend d	< 0.001	0.001	0.001
Healthy eating behaviors (≥=6 vs <6)			
<6	1.000 (reference)	1.000 (reference)	1.000 (reference)
≥=6	0.161 (0.039, 0.666)	0.172 (0.041, 0.716)	0.174 (0.042, 0.725)
P for trend d	0.012	0.016	0.016
Healthy eating behaviors (≥=7vs <7)			
<7	1.000 (reference)	1.000 (reference)	1.000 (reference)
≥=7	0.145 (0.015, 1.418)	0.136 (0.013, 1.435)	0.130 (0.013, 1.318)
P for trend d	0.097	0.097	0.084

a Model 1: crude

b Model 2: Adjusted for sex, age, annual family income, BMI,father and mother's education (primary and below, junior high, high school, or junior college and above).

c Model 3: Model 2+sleep duration and sleep quality.

d Adjusted data are expressed as odds ratio (95% confidence intervals).

Supplement table 21 Multivariate logistic regression of the risk of depressive symptoms according to sugar-sweetened beverages consumption

(Chinese children and adolescents)

	Model 1 a	Model 2 b	Model 3 c
Sugar-sweetened beverages (none vs ≥once/week)			
≥once/week	1.000 (reference)	1.000 (reference)	1.000 (reference)
None	0.530 (0.302, 0.931)	0.512 (0.285, 0.917)	0.512 (0.286, 0.921)
P for trend d	0.027	0.024	0.025

a Model 1: crude

b Model 2: Adjusted for sex, age, annual family income, father and mother's education (primary and below, junior high, high school, or junior college and above).

c Model 3: Model 2+sleep duration and sleep quality.

d Adjusted data are expressed as odds ratio (95% confidence intervals).

Supplement table 22 Multivariate logistic regression of the risk of depressive symptoms according to fry food consumption (Chinese children and adolescents)

	Model 1 a	Model 2 b	Model 3 c
fry food (none vs ≥once/week)			
≥once/week	1.000 (reference)	1.000 (reference)	1.000 (reference)
None	0.408(0.242,0.687)	0.385(0.225,0.659)	0.388(0.227,0.665)
P for trend d	<0.001	<0.001	<0.001

a Model 1: crude

b Model 2: Adjusted for sex, age, annual family income, father and mother's education (primary and below, junior high, high school, or junior college and above).

c Model 3: Model 2+sleep duration and sleep quality.

d Adjusted data are expressed as odds ratio (95% confidence intervals).

Supplement table 23 Multivariate logistic regression of the risk of depressive symptoms according to fruits consumption (Chinese children and adolescents)

	Model 1 a	Model 2 b	Model 3 c
Fruits (none vs ≥once/day)			
None	1.000 (reference)	1.000 (reference)	1.000 (reference)
≥once/day	0.385(0.217,0.685)	0.458(0.246,0.852)	0.472(0.252,0.885)
P for trend d	<0.001	0.014	0.019

a Model 1: crude

b Model 2: Adjusted for sex, age, annual family income, father and mother's education (primary and below, junior high, high school, or junior college and above).

c Model 3: Model 2+sleep duration and sleep quality.

d Adjusted data are expressed as odds ratio (95% confidence intervals).

Supplement table 24 Multivariate logistic regression of the risk of depressive symptoms according to Vegetable consumption (Chinese children and adolescents)

	Model 1 a	Model 2 b	Model 3 c
Vegetable (none vs ≥once/day)			
None	1.000 (reference)	1.000 (reference)	1.000 (reference)
≥once/day	0.673(0.282, 1.610)	0.651(0.267, 1.585)	0.673(0.275,1.643)
P for trend d	0.374	0.344	0.384

a Model 1: crude

b Model 2: Adjusted for sex, age, annual family income, father and mother's education (primary and below, junior high, high school, or junior college and above).

c Model 3: Model 2+sleep duration and sleep quality.

d Adjusted data are expressed as odds ratio (95% confidence intervals).

Supplement table 25 Multivariate logistic regression of the risk of depressive symptoms according to breakfast consumption (Chinese children and adolescents)

	Model 1 a	Model 2 b	Model 3 c
Breakfast(none vs ≥once/week)			
None	1.000 (reference)	1.000 (reference)	1.000 (reference)
≥once/week	0.236(0.136,0.411)	0.249(0.140,0.445)	0.252(0.141,0.450)
P for trend d	<0.001	<0.001	<0.001

a Model 1: crude

b Model 2: Adjusted for sex, age, annual family income, father and mother's education (primary and below, junior high, high school, or junior college and above).

c Model 3: Model 2+sleep duration and sleep quality.

d Adjusted data are expressed as odds ratio (95% confidence intervals).

Supplement table 26 Multivariate logistic regression of the risk of depressive symptoms according to fast food consumption (Chinese children and adolescents)

	Model 1 a	Model 2 b	Model 3 c
Fast food(none vs ≥once/week)			
≥once/week	1.000 (reference)	1.000 (reference)	1.000 (reference)
None	0.400(0.237,0.675)	0.388(0.226,0.665)	0.393(0.229,0.675)
P for trend d	<0.001	<0.001	<0.001

a Model 1: crude

b Model 2: Adjusted for sex, age, annual family income, father and mother's education (primary and below, junior high, high school, or junior college and above).

c Model 3: Model 2+sleep duration and sleep quality.

d Adjusted data are expressed as odds ratio (95% confidence intervals).

Supplement table 27 Multivariate logistic regression of the risk of depressive symptoms according to processed food consumption (Chinese children and adolescents)

	Model 1 a	Model 2 b	Model 3 c
Processed food(none vs ≥once/week)			
≥once/week	1.000 (reference)	1.000 (reference)	1.000 (reference)
None	0.559(0.327,0.957)	0.547(0.314,0.953)	0.546(0.312,0.953)
P for trend d	0.034	0.033	0.033

a Model 1: crude

b Model 2: Adjusted for sex, age, annual family income, father and mother's education (primary and below, junior high, high school, or junior college and above).

c Model 3: Model 2+sleep duration and sleep quality.

d Adjusted data are expressed as odds ratio (95% confidence intervals).
